# Advancing collaboration in health professions education in the general practice domain, developing a national research agenda

**DOI:** 10.1007/s10459-024-10340-4

**Published:** 2024-05-27

**Authors:** Esther de Groot, Marianne Mak-van der Vossen, Irene Slootweg, Meryem Çorum, Anneke Kramer, Jean Muris, Nynke Scherpbier, Bart Thoonen, Roger Damoiseaux

**Affiliations:** 1 Julius Center for Health Sciences and Primary Care, University Medical Center Utrecht, Utrecht University, Utrecht, The Netherlands; 2 Department of General Practice, and Amsterdam Public Health research institute, Amsterdam UMC, Amsterdam, The Netherlands; 3 Department of Public Health and Primary Care, Leiden University Medical Center, Leiden, The Netherlands; 4 Department of Family Medicine, CAPHRI Care and Public Health Research Institute, Maastricht University, Maastricht, The Netherlands; 5 Department of Primary and Long-term Care, Groningen Medical Center, Groningen, The Netherlands; 6 Department of Primary and Community Care, Radboud UMC, Nijmegen, The Netherlands

**Keywords:** Inter-institutional research collaboration, Q-methodology, General Practice, Research agenda

## Abstract

**Background:**

Health professions education (HPE) research in the General Practice domain (GP-HPE) is vital for high-quality healthcare. Collaboration among GP-HPE researchers is crucial but challenging. Formulating a research agenda, involving stakeholders, and fostering inter-institutional collaboration can address these challenges and connect educational research and practice.

**Methods:**

We used Q-methodology to explore perspectives on GP-HPE research of participants from all Dutch postgraduate GP training institutes. Participants individually sorted statements based on the relevance of future GP-HPE research for educational practice. Data analysis comprised inverted factor analysis, rotation, and qualitative interpretation of configurations of all statements. The National Meeting on Educational Research took a participatory approach.

**Results:**

We included 73 participants with diverse involvement in GP-HPE research. We identified five distinct perspectives, each representing a research focus area for developing and innovating GP education: the clinician scientist, the socially engaged GP, the specific GP identity, the GP as an entrepreneur, and the GP engaged in lifelong learning.

**Discussion:**

The resulting five perspectives align with General Practice hallmarks. Q-methodology and a participatory approach facilitated collaboration among stakeholders. Successful inter-institutional collaboration requires a common goal, neutral leadership, participant commitment, regular meetings, audit trail support, process transparency, and reflexivity. Future research should address evidence gaps within these perspectives.

**Conclusion:**

Using Q-methodology turned out to be valuable for compiling a national research agenda for GP-HPE research. The research process helped to cross boundaries between researchers in different institutions, thus putting inter-institutional collaborative advantage center stage. Our approach could provide a conceivable procedure for HPE researchers worldwide.

**Supplementary Information:**

The online version contains supplementary material available at 10.1007/s10459-024-10340-4.

## Introduction

### Background

Health professions education (HPE) and the development and innovation thereof through research are increasingly vital for high-quality healthcare and cure. For the subdomain of the General Practice setting, Grierson and Vanstone (2021) identify four hallmarks that make this domain advantageous to the purpose of HPE research: the relational care, the community-based approach, the complexity and uncertainty of the work, and the breadth of knowledge and skills (Grierson & Vanstone, 2021). Furthermore, general practitioners (GPs) are expected to play an ever-increasingly important role within the healthcare system. As a result, the education of GPs—and the evidence base for this education—will become even more critical in the following decades (Kidd, 2013). In GP-HPE, complex issues are ubiquitous and—due to the transitions in healthcare—dynamic and subject to change, bringing a high risk of research fragmentation.

Hence, collaboration among GP-HPE researchers is essential. Inside and outside of the HPE domain, research collaboration contributes to more impact and higher quality of research (Kyvik & Reymert, 2017), because of a critical mass of people with complementary skills and expertise (Kenna & Berche, 2011). Presumably, research collaboration also helps to answer the need for more theoretically supported research and theory building (Gordon & Cleland, 2021; Samuel et al., 2020). Research collaboration also creates opportunities for the division of labor, structural exchange of expertise, and opportunities for focus when applying for external funding.

In contrast with the assumed advantages of collaboration and high-quality research demands, research collaboration does not happen automatically; this is also the case in HPE research (Stadler et al., 2019). Many studies on research collaboration, often using social network approaches to show co-authorship practices (Kezar, 2005; Kyvik & Reymert, 2017; Vermond et al., 2022), have indicated that research collaboration only happens under certain conditions. Besides, HPE research asks for *interdisciplinary* collaboration, which is often even more challenging because researchers must bridge boundaries between disciplinary paradigms (Mäkinen et al., 2020). Furthermore, HPE research falls in the social science domain, where collaboration takes longer to develop (Lewis et al., 2012).

In many countries, to our knowledge, researchers who produce the evidence base for GP education and lifelong learning are located across various institutes and they lack structured collaboration. The literature on inter-organizational and inter-institutional (research) collaboration confirms that hierarchical organization arrangements must be more suitable in those contexts (Hardy et al., 2003). Accordingly, although virtual communication offers new opportunities, research collaboration is likely to develop only slowly in networks of researchers working in different institutes at the national or international level. In GP-HPE research, groups are small, and only limited financial resources are available (Gruppen & Durning, 2016). Financial resources are important because the quality of research is related to funding (Reed et al., 2007). In sum: research collaboration is incredibly challenging in an inter-institutional context.

Responding to the challenges of increasing demands for evidence and limited financial resources that jeopardize the quality of GP-HPE research, formulating collective research goals in a shared research agenda is a logical step to starting successful collaboration (Stadler et al., 2019). However, the way to develop a research agenda in this domain has yet to be apparent. The reasons for this are manifold. Some of those reasons apply specifically to the General Practice domain; others are valid for HPE research in general. Two of these challenges are especially relevant to this study.

First, GP-HPE research combines two broad research domains: HPE and General Practice. HPE is a multidisciplinary field and thus builds upon a large diversity of research traditions. General Practice is also a wide-ranging and dynamic domain. As a result, two often applied approaches for establishing research agendas, identifying gaps by scanning the literature and assessing impact, do not suffice. A broad-ranging literature review is complicated and very time-consuming. Agreeing on priorities based on impact is tough because opinions about research impact are often derived from the medical domain and differ from opinions on this topic within the HPE domain (Varpio et al., 2020).

Second, as Worley and colleagues (2014) stated in a critical reflection on priority-setting exercises for HPE research: only asking stakeholders in postgraduate institutes (teachers and developers) what is necessary is insufficient to formulate a sustainable research agenda (Worley & Schuwirth, 2014). Agendas have to bridge “the gap between those who know the health professional education literature and contribute to it, and those who understand the needs and desires of the practical context” (Worley & Schuwirth, 2014, p. 1034). Their worries align with the tensions identified by Albert and colleagues (2007): Should HPE be practical and problem-oriented or theoretically oriented toward contributions to the international knowledge base in HPE research? (Albert et al., 2007) In an applied research domain, researchers have to cross boundaries between practice and research. Boundary objects facilitate boundary-crossing which could help to unify different viewpoints between producers and consumers of scientific knowledge. In boundary-crossing theory, a boundary object may take different shapes and could “be used for understanding how coordination and collaboration across boundaries is facilitated” (Karlsson et al., 2020, p. 242).

As a response to these challenges, there is a need for a methodology that pays attention to two specific aspects that have previously not been considered when selecting a methodology for developing a research agenda: (1) reflexivity on the collaboration process between representatives who work in institutes with distinct organizational cultures and (2) starting with themes instead of priorities to prevent each participant contributing their own personal -established- expertise. The Q-methodology offers the possibility to acknowledge reflexivity and innovative interpretations while developing a research agenda. Until now, the Q-methodology has not been applied with the considerations for future collaboration in mind. While Yau et al. used Q-methodology, they did not highlight future collaboration, as they focused on understanding why participants prioritize their viewpoints (Yau et al., 2021).

In the Netherlands, where GP postgraduate training institutes are often embedded within Academic Medical Faculties, thematic agendas of educational research have traditionally been established by each postgraduate training center separately or sometimes they have not been established at all. We argue that collectively exploring the perspectives of various stakeholders contributes to the first necessary stage for research collaboration between researchers from different institutes (Kezar, 2005): Building commitment to collaborate. The various perspectives provide opportunities for future research, filling gaps in the GP-HPE domain, building on existing research experience, and encouraging cooperation between researchers, thus increasing research impact (Kyvik & Reymert, 2017). Finally, such collaboration provides research opportunities that are only possible with a national approach. With collaborative advantage (Vangen & Huxham, 2003), research groups manage their collaborations to achieve things that could not be attained by any of the groups alone. Trusting relationships are important in the theory of collaborative advantage. Thus far the research groups were competing with one another, realizing the potential for collaborative advantage needs to be built gradually. In the long run, this mapping of perspectives and reflections on colliding viewpoints allows the positioning of the GP-HPE research domain in the broader HPE research landscape, both nationally and internationally.

## Research aim

The overall objective of this project was to foster collaboration between universities within the GP-HPE research domain. The first step to achieve this goal was the development of a research agenda through conducting a nationwide Q-methodology study (Watts & Stenner, 2012) as this methodology, based on boundary-crossing theory, was assumed to help in bridging educational research and practice.

Our research question was: What are the different perspectives of learners, clinical and non-clinical educators, curriculum developers, and researchers on topics crucial for conducting HPE research in the domain of General Practice in the Netherlands?

This study aimed not only to describe the perspectives that fit a future research agenda but also to make our collaboration process transparent. Describing and discussing the process is internationally relevant to others contemplating social innovations toward inter-institutional research through the lens of collaborative advantage theory and boundary-crossing theory.

## Methods

### Study design

We designed an empirical single case study using Q-methodology (Grijpma et al., 2021; Watts & Stenner, 2012) to elucidate the perspectives of informed participants on HPE research in the General Practice domain. The Q-methodology fits our research goal as it systematically investigates a subjective topic, aiming to distinguish different perspectives on that topic (Churruca et al., 2021; Lundberg et al., 2020). These aspects were considered crucial for fostering collaboration. Participants conducted a Q-sorting, in which they individually used a fixed grid to sort a set of previously constructed statements based on literature review and expert opinions. Using both statistical and qualitative analysis, Q-sorting combines “the richness of qualitative data with the rigor of statistical analysis” (Churruca et al., 2021, p. 7). Finally, the practicalities of this method were suitable for our purpose because the Q-sort methodology requires only a limited number of participants, usually about 40–60 (Churruca et al., 2021; Watts & Stenner, 2012). The purpose is to identify the existence of certain viewpoints, generalization to the larger population is not the aim (Watts & Stenner, 2012).

### The Dutch context

Eight Dutch GP postgraduate training institutes, each affiliated with a different university, collaborate in *Huisartsopleiding Nederland* (HN), where organizational and educational initiatives for GP training are shared. Up till now, HN has not been coordinating GP-HPE research. A group of educational researchers united under the flag of HN aimed to set the next step in national collaboration of education research by establishing a shared effort to create a national research agenda in the Netherlands. The training institutes are responsible for the three-year postgraduate training of GPs. The statements in the Q-methodology concourse concerned the continuum of GP education in which students transition from undergraduate to postgraduate education. At a national level around 700 trainees/yr are entering GP training. Academic development for GPs after graduation is mandatory.

### Reflexivity and paradigm

For this study, we took a constructivist stance, meaning that we consider knowledge as actively being constructed based on the experiences of participants and researchers alike and co-created as the product of their interactions and relationships (Romm, 2013). Based on our constructivist stance, we acknowledge that participants and researchers collectively co-created the findings of this study: The results originate from their shared knowledge and day-to-day experiences. To inform the readers about the knowledge and experiences that the authors themselves brought into this study, we present the following information to our readers. All authors are medical educators and scientists in HPE. All authors were involved in the whole study. A subset of the author group conducted the qualitative data analysis. Their background is as follows: EG is a molecular biologist who turned into an educational researcher, MM is a medical doctor who works in HPE research, IS is an associate professor in HPE research, RD is a GP, head of a postgraduate training institute and a professor, and MÇ was a student in the learning sciences. The research question for this study originated from our own practical research experience. We kept an audit trail (Akkerman et al., 2008) that we regularly discussed with the full research team to look at our contribution to the study process.

### Data collection

The procedure for data collection in this study comprised three steps:

- The concourse: Creating an expert group and identifying topics for the Q-sorting
- Participants selected for the Q-sorting
- Q-sorting and interviewing

#### Step 1 The concourse: creating an expert group and identifying topics for the Q-sorting

A first start has been to create an expert group in this domain, of which most members are authors of this paper or mentioned in the acknowledgments. This group of experts generated a list of statements for inclusion in the next step of the process, the Q-sorting exercise. We started with a broad list of Q-statements collected in an agenda-setting exercise for a national HPE research agenda in Taiwan (Yau et al., 2021). All experts judged this list independently and assessed the relevance of each statement for the Dutch situation. Besides, the experts indicated which topics were irrelevant for an agenda in our domain of learning and teaching within (continuing) education for General Practice. In addition, the experts added statements to the list based on their expertise or on sources that they were free to consider for further inspiration. The experts got online access to a list of sources that were related to fields that are relevant to our domain, for example, reports on the future of General Practice, papers on opportunities for GP-HPE research, policy documents on medical education research in the Netherlands, and Dutch policy reports on education for primary care.

An overview of the diverging opinions on the relevance of some statements was collected in Excel. We omitted the topics for which less than five respondents chose “relevant for the GP-HPE domain”. Based on the experts’ feedback, it was decided that the level of aggregation of the items in the original list was not balanced. Some statements (such as “preparedness for future practice”) were considered overarching themes for further interpretation but were not suitable for inclusion in the set of Q-sortingstatements. Next, in the second round, the retained statements and new topics were circulated. In this round, the group of experts, independent of one another, indicated for which topics no new research was considered necessary based on the available existing research already. No topics were eliminated in this round (Table 1).

**Table 1:** Development of the concourse in number of statements

	Nb of statements
Original concourse	67
Left out because more than 5 participants judged these to be irrelevant for the General Practice context	11
Left out because the level of aggregation was different from the other items and therefore not specific enough	12
Left out because these statements were only applicable to researchers and not to practitioners (e.g. funding of research)	5
New items added based on sources	25
Final amount of statements:	64

#### Step 2 Participants selection for the Q-sorting

The following stakeholders were asked to participate in the Q-sorting.

- Teachers in GP postgraduate education as well as GP undergraduate education
- Supervisors
- Students (in GP postgraduate education)
- Medical education researchers within the General Practice domain
- Developers of educational material for the postgraduate training institute
- Management of the postgraduate training institute.

In a Q-sorting exercise, there is no need for an equal number of representatives for each role. We checked (see results section) that all stakeholders were represented in the Q-sortings. The rationale for this was that not every participant with a specific role (e.g. teacher) has the same perspective on the topic. In Q-methodology, it is especially relevant to have a broad mix of participants with various perspectives on the topic under investigation. A total number of 73 participants were included in the data collection sessions (Q-sorting). From each institute, about nine people took part.

#### Step 3 Q-sorting and interviewing

A pilot session was organized to assess how long sorting would take and if the instructions were clear. After this pilot, no new statements were added, and the instruction was changed slightly. Next, sessions were organized in each institute with a diverse mix of students, teachers, researchers, developers, and managers. For an overview of who participated in which institute, see Table 3 in the results section. The Q-sorting was done individually, but to allow for questions, one or two researchers (IS, MM, EG, or MÇ) were present in each session. They supported the respondents by holding brief interviews after the sorting in which they asked respondents to clarify why they sorted the statements in this particular manner. Participants were also allowed to provide additional topics for which they could not find enough evidence in the literature.

Each participant received a set of Q-sorting statements including explanations for each statement. Before the sorting exercise, they were given some time to read an introductory text (in Dutch, see Appendix for the translated version). In the pilot session, participants indicated that it was difficult to separate the idea “it is important in GP training” from “it is important to research this topic”. To help them keep the goal in mind, every participant put a chart with the research question on their table while doing the sorting session.

As a first step, participants were invited to compile three stacks: (1) important to include in a national research agenda, (2) not important, and (3) neutral. Next, participants ranked laminated statement cards using a standard Q-grid with a 13-point distribution (− 6 to + 6). The Q-grid had been designed so that respondents must make choices because there is only a limited number of cells under the heading “very important” or “not at all important”. During the sorting process, participants were not allowed to discuss their sorting with other respondents, as it was not a consensus-reaching session. Immediately after sorting, each participant was asked briefly about their sorting result (see Appendix). Not all questions were asked for each respondent to keep the burden for the respondents low, and a few had to leave early. The most important points about their reasoning were written down. After each session, a photograph was taken of each sorting, the overall sorting was copied on an empty grid, and the sorting from all participants was entered into an Excel sheet.

### Data analysis

Data analysis in this study comprised three additional steps:

- Inverted factor analysis
- Rotation of the factors
- Factor interpretation

For analyzing the quantitative data, the software tool KADE was used (Banasick, 2019).

#### Step 4 Inverted factor analysis

The first step in the quantitative analysis was to perform an inverted factor analysis, resulting in a correlation matrix showing which participants sorted the statements in similar configurations (Watts & Stenner, 2012). The next step was a centroid factor analysis. To assess how many factors to keep in this stage, we considered the Eigenvalues (EV). Eigenvalues provide information on the commonality concerning each factor instead of each participant (Watts & Stenner, 2012, p. 104). Based on the recommendation by Watts and Stenner (2012), we kept all factors with values higher than one (in our case, the EV ranked from 10.9 to 1.7). We calculated whether the total variance explained was higher than 35% (with eight factors, the total explained variance was 44%). Furthermore, we considered the Scree plot. If we followed the Scree test, we would keep just three factors, but then the explained variance would be below 35%, and additionally, in the next steps, many participants would be left out. Watts and Senner recommend (page 110) keeping a larger number of factors in this stage if the quantitative tests are inconclusive (Watts & Stenner, 2012). Therefore, we kept eight factors.

#### Step 5 Rotation of the factors

Next, a Varimax rotation was performed. The rotation is to identify any participant “that closely approximates the viewpoint of that particular factor” (Watts & Stenner, 2012, p. 130). Within the KADE tool, different levels of significance could be chosen, with a smaller level of significance (*P*-value), as in this step we kept as many participants as possible. However, with a larger *P*-value, the resulting factors (we call them in the result section “perspectives”), based on a smaller number of participants, would become easier to interpret. In comparing the effects of different *P*-values and our wish to keep sufficient participants in the final analysis, we decided on a *P*-value of 0.05.

#### Step 6 Interpreting the factors

The principal researchers interpreted the holistic ordering of all the statements on the grid by an anticipated “ideal” respondent who sorted the statements according to that perspective. In addition, we studied the crib sheets for each factor in which distinguishing statements (in a positive or negative sense) were depicted. In interpreting these “ideal” sorts and the cribs sheets, we discussed among the researchers where we returned to the brief interviews if necessary. We wrote down a description indicating what perspective someone who sorted the statements in that way would consider necessary in GP-HPE research.

When the factors had been described, they were presented to the entire team of experts asking for their feedback. One of the main points of feedback was that it was necessary to clarify which educational priorities were related to each perspective. In the next round of editing by the core group of researchers, we returned to the factor arrays and the interviews and adjusted the descriptions to clarify the educational relevance of each perspective.

### Ethical consent for the Q-sorting

The Medical Ethics Review Committee (METC) of the University Medical Center Utrecht declared that this research did not require formal approval as it was not subject to the Dutch Medical Research Involving Human Subjects Act (WMO). Despite this waiver, we informed every participant about the purpose of the study, where the data was stored securely, and how they could opt out during the session. This information was distributed before the session. Participants gave written informed consent.

## Results

### Participant characteristics

Table 2 summarizes the distribution of people with certain levels of involvement with GP-HPE research. It indicates that our set of participants was diverse concerning educational research experience.

**Table 2:** Manner of involvement from participants to the sections

	Nb of times
Participated in educational research	32
Performed educational research	30
Applied results of educational research in the design of my teaching activities	46
Applied results of educational research in the way I teach	44
Was involved differently in education research, being: …	30

*Participants were allowed to choose more than one manner of involvement; therefore the rows in this table are not exclusive

In the brief interviews after the Q-sorting, participants mentioned a few topics they felt were lacking. The researchers considered these topics to be sub-topics of statements that were part of the concourse already. A few participants mentioned that their sorting was influenced by their ideas about research that had been done in earlier years, for example about clinical reasoning or selecting. For educational practice, more in-depth knowledge about those topics was not required anymore. Participants also shared their reflections on the process of Q-sorting. They expressed that the statements were all relevant and that it took work to prioritize, which involved forced choice of statements.

### Factor development

The IFA resulted in eight factors. After Varimax rotation, a solution of four or five factors appeared to be feasible, based on EV, using the Kaiser Guttman criterion. With a solution of four or five factors, we kept factors with an EV higher than 1. For both factors, we compared the resulting crib sheets and the number of participants that would be left out if we used different P-values. Based on a qualitative interpretation of the crib sheets for four or five factors respectively, we decided with the group of researchers (IS, MM, EG, RD, MÇ) that keeping five factors, with a P-value of 0.05, did justice to the results the most (Table 3).

**Table 3:** Factor score correlations

	Factor 1	Factor 2	Factor 3	Factor 4	Factor 5
Factor 1	1	0.17	0.26	0.23	0.27
Factor 2	0.17	1	0.28	0.34	0.03
Factor 3	0.26	0.28	1	0.17	0.09
Factor 4	0.27	0.03	0.09	1	0.18
Factor 5	0.23	0.34	0.17	0.18	1

*Based on these scores and a group discussion about possible interpretations, we concluded that keeping five factors was a good decision. These correlations are calculated in the factor development phase. The final interpretations are the same as the described perspectives which make explicit five areas of research; research area one: The clinician-scientist up until research area five: The GP engaged in lifelong learning. The order of the factors is presented in line with these final interpretations. The factor originally the fourth factor was placed at the end because it has a slightly different color than the other factors (perspectives)

With the final number of factors, there were no consensus statements. Consensus statements are statements that all respondents agree upon the location in the grid and thus do not distinguish between any pair of factors.

### Factor interpretation: five perspectives

Each factor from the first steps represents the perspective of a group of participants on the question of what scientific research is particularly needed to improve and renew research on learning and education within the continuum of GP education. These perspectives explicit five distinctive areas of research, together forming the proposed research agenda. The title of each research area describes the purpose of the desired research on learning and education: (1) The clinician scientist, (2) The socially engaged GP, (3) The specific GP identity, (4) The GP as an entrepreneur, (5) The GP engaged in lifelong learning.

## Research area 1: The clinician-scientist

This research area focuses on the training of GPs as medical experts who use new knowledge in the consulting room, in conjunction with previously acquired expertise, scientific knowledge, and the wishes of the patient.

Research into how the learner acquires specific General Practice expertise and learns to apply it in the workplace is appropriate for this research area. General Practice knowledge is specific to the field of General Practice, and a doctor with a basic medical qualification who has been trained in a hospital will possess such knowledge to a limited extent at the most. A GP’s clinical reasoning is based on the epidemiology of diseases in primary care. In this context, symptoms have a different predictive value than in a hospital, and this requires the GP to take a different approach, as it is more difficult to make the right decision with less information. Attention to patient safety is important in this research area, as it can be negatively affected by both undertreatment and overtreatment. This research area mainly concerns individual learning processes, such as experiential learning.

Knowledge of how GPs develop medical thinking and action is essential for the targeted training of learners to become clinician-scientists. *“Evidence-based care is most important for the quality of the GP.”*

## Research area 2: The socially engaged GP

This research area concerns the training of GPs who actively contribute to the debate on health matters that are of importance to society and can help bring about the necessary changes.

Suitable for this research area is research focusing on how learners are trained to become professionals who see developments in society—where relevant to healthcare—as part of their profession and are capable of introducing these changes into professional practice. The GP learns to handle different views, both on collective patient interests and on the organization of healthcare. This concerns how the healthcare process is arranged, and encompasses matters like continuity of care, community-based work, healthcare innovation, running a practice, and interprofessional collaboration. It also concerns the development of autonomy and major developments occurring in healthcare, such as Planetary Health which is an emerging field addressing the interrelation between humans and ecosystems. Educational processes that focus on learning together—with the patient, with other medical professionals and allied health professionals, and with disciplines outside healthcare—are suited to this research area.

Knowledge of how to support learning and development is needed, to improve the training of GPs who can play an influential role in the debate on developments in society that have implications for healthcare. *“We must take care of social and professional education and be concerned about the Planetary Health, otherwise human civilization will be in danger.”* And less outspoken: “*The development of the profession, the shortages in the care sector and the importance of new forms of work.”*

## Research area 3: The specific GP identity

This research area draws attention to the training of GPs as generalists with the competencies required in the role of a GP in the broad sense.

Suitable for this research area.is research focusing on the way learners can be trained to become professionals with a wide range of employable skills, who see the patient as a whole and can take responsibility even in the face of residual uncertainty. A specific and inherent aspect of General Practice is how GPs deal with uncertainty. They need to accept uncertainty to act in the interests of patient dignity, for example by performing worthwhile, targeted diagnostic tests and preventing overtreatment. This cannot always be secured with better evidence or better clinical reasoning. Elements requiring attention include ensuring that learners are aware of different views, learn how to weigh up ethical and moral considerations, and acquire excellent communication skills. This learning process starts when they are studying and remains important throughout their professional practice. Learning to deal with uncertainty requires resilience, and a safe and supportive learning environment is a constant prerequisite. The main concern of this research area are learning processes that are being studied at the level of the individual. Three domains of purpose at this level are socialization, subjectification, and qualification (Biesta, 2020).

Knowledge of the subjects addressed in this research area is important for the training of GPs who see dealing with uncertainty as an inherent part of their professional identity. *“The breadth of the profession and uncertainty is a major theme for GP trainees.”* And less outspoken*: “Teacher development is essential.”*

## Research area 4: The GP as an entrepreneur

This research area addresses the training of innovative entrepreneurs for the changing healthcare landscape, GPs who put the knowledge of system changes, new technology, and multimedia applications into practice.

Research into learners with an entrepreneurial attitude taking on a role in organizational change in GP care, which goes beyond traditional business ventures, is suitable for this research area. The aim is subjectification, which gives learners room to link their roles as entrepreneurs, innovators or change agents with their own personal and professional ambitions. Subjects such as recruitment and selection, remediation, transitions in healthcare, and more flexibility in education are also important in this research area. Learners seeking challenges in terms of innovation in the organization of healthcare are thus able to learn in their own way how to fulfill these roles. This research area encompasses innovative social learning processes.

Knowledge of the development of the role of entrepreneur in General Practice is relevant to prepare learners for changes in the organization and provision of GP care. *“Role of apprenticeship about the longitudinal learning process of the GP trainee.”* And less outspoken: “*Quality improvement in healthcare in the broad sense.”*

## Research area 5: The GP engaged in lifelong learning

This research area draws attention to the theoretical foundations of learning and training. Research on issues associated with learning and learning environments in the context of General Practice, designed to provide better understanding and prompt innovation, is suitable for this research area. The focus is on research into the design of the learning environment in a broad sense, the contribution that patients make to learning processes, bridging gaps between different parts of the training continuum, and fostering lifelong learning in the workplace. This research area also draws specific attention to the knowledge of professionalization among teachers and trainers.

Conceptual knowledge of learning processes and learning environments is important to ensure that research aligns with international developments in medical education. A stronger theoretical base strengthens the quality of education research: *“All learning is lifelong learning.”*

### Findings from reflections on research collaboration

- The scientific approach to setting the research agenda can only be appreciated through a thorough reflection on how the agenda has been created in a co-creation process. We reflected through the lens of collaborative advantage (Vangen & Huxham, 2003). Our trajectory started with a modest goal of setting an agenda with a few broad research areas instead of trying to establish priorities in an inter-institutional context where nobody is having the power to decide in a top-down manner. This helped us to develop trusting attitudes between researchers competing with one another earlier. Trust was considered necessary for more ambitious collaboration in later stages. Our reflection on the notes of the National Meeting on Research in Education (which we call “La OvO”) revealed the following: Between November 2021 and January 2023, there have been eight digital meetings of at least 90 min, with the principal HPE investigators of all eight GP training institutes. These meetings led to an open co-creation with the entire La OvO group and great alignment.
- A smaller core group (RIME) was formed from this larger group. This group has been mandated and trusted to prepare and oversee the process for all members of La OvO. This allowed for an increase in creativity, energy, and speed as they could meet in person more easily.
- The La OvO meeting—chaired by an independent chairperson (AK)—could make decisions, adapting the RIME group’s proposals to all local wishes and ambitions. All eight GP training institutes had an equal say in deciding on content and process.
- As described in the design, all research process steps were prepared by the RIME group and submitted to the La OvO meeting for decision-making. This approach led to transparency in decision-making on the meso-level and the formation of appropriate expectations.
- Data collection in the institutes was the responsibility of the La OvO members involved in the co-creation, which radiated down to the institutes at the micro level.
- The chairman of the La OvO group (RD) brought in updated information from the funding agency at every meeting. That macro-level contact gave clarity on the deadline and end goal.

## Discussion

The purpose of this study was to foster collaboration between universities within the GP-HPE research domain where—the development of—a research agenda could guide future research in GP-HPE. The resulting Dutch national research agenda is based on the perspectives of those directly involved in GP education and educational research regarding certain topics in GP education. We found five different perspectives on topics in GP-HPE that need further research: (1) The clinician scientist, (2) The socially engaged GP, (3) The specific GP identity, (4) The GP as an entrepreneur, and (5) The GP engaged in lifelong learning.

### What this study adds to the literature

Grierson states that General Practice has four hallmarks that make it advantageous to the purpose of HPE research: (a) relational care, (b) the community-based approach, (c) the complexity and uncertainty of the work, and (d) the breadth of knowledge and skills (Grierson & Vanstone, 2021). Interestingly, our participants’ perspectives align closely with these hallmarks, emphasizing the need for research on effectively teaching them. Specifically, our participants called for educational evidence for hallmarks b, c, and d. This may be because our participants felt that the teaching of relational care, including communication and empathy, has already been adequately explored in our Dutch context.

Our study also revealed a perspective not explicitly mentioned as a hallmark of General Practice by Grierson. In our study, this topic turned out to be considered worthy of further exploration in GP-HPE research: GP’s entrepreneurship. While back in the seventies the picture of the GP was the head of a one-man practice, over time it has broadened into new ways of organizing generalism in the healthcare system, including diverse professionals collaborating in local, regional, and (inter)national networks. Moreover, our findings suggest that research into theoretical concepts (our perspective 5) is needed to underpin the more applied perspectives 1–4, which is in line with Samuel’s call for more theoretically supported research in HPE (Samuel et al., 2020).

In the introduction, we claimed that even though setting research priorities is vital to formulate research questions that truly make a difference and are in tune with the needs of stakeholders, for inter-institutional collaboration in an interdisciplinary or multidisciplinary research domain to become successful, starting with priority setting right away might not be the best approach. Below, we reflect on the use of Q-methodology and inter-institutional research collaboration in line with this.

In the process of our work, the choice of the Q-methodology was pivotal. Q-methodology brings to the fore how different items “are evaluated in relation to each other” (Leidig et al., 2022, p. 443). For educators, developers, and researchers in General Practice, who are primarily interested in the broad perspective of their profession, such evaluations are better suited than questionnaires in which each item is judged independently, and all items could be rated as “very important”. The participatory approach was also valuable for bridging the gap mentioned by Worley and Schuwirth (2014) as in the Q-sorting, a mix of different participants took part, without the need for an equal number of participants from each subgroup. The Q-methodology does not assume that all teachers, for example, think the same, and subgroup analyses are possible but not necessary. This was advantageous for our project, because in our setting the number of researchers is much smaller than the number of teachers. On a more pragmatic note, researchers wanting to develop an agenda and simultaneously lay the foundation for more intense research collaboration need a relatively accessible and low-cost approach. For the Q-methodology, small sample sizes are sufficient (Watts & Stenner, 2012).

As in the analysis, the holistic ordering of all the statements and analysis of the ordering within the Q- methodology has advantages compared with other methods for research agenda setting. Respondents must consider every item for a position in the Q-grid (Leidig et al., 2022) and order the items individually, not in a consensus meeting with each institute. The Q-sorting process may be seen as a boundary object (Star, 2010), as a few broad research areas are sufficiently helpful to serve producers and consumers of research findings. As such, this method was perceived to be valuable for building commitment to teamwork between institutes that earlier had to compete with each other in the infrequent grant calls. In this first step, the boundary object “Q-sorting” did not yet play a major role in helping different groups understand each other's perspectives better. In line with our small-steps approach, advocated by collaborative advantage theory, for the future, we consider organizing follow-up meetings to be useful.

What helps to support the collaborative development of a national research agenda between researchers from research groups in different universities? Collaborative development of a national research agenda between researchers from research groups in different universities can occur if certain conditions are fulfilled. The strategies that we consider crucial to success include a common goal, frequent online meetings, a neutral president, participant commitment, adequate support for maintaining an audit trail, transparency about the process, and participants’ reflexivity (Olmos-Vega et al., 2022). Even though most papers on inter-organizational or inter-institutional collaboration look at a higher unit of analysis, we argue that differences —for example in culture—at different institutes should be made explicit when considering research collaboration. As Karlson et al. (2020) underlined, even though their work is about inter-organizational collaboration in healthcare processes, the concept of boundary objects is important. In our work, boundary objects thus play a role in two different ways: (1) the Q-sorting process as a bridge between consumers and producers, and (2) the process of constructing a collective research agenda in co-creation may be considered a boundary object. This process helped build the bridge between boundaries between different academic institutes, facilitating research collaboration. Future research in both boundary object roles of the research agenda development is necessary.

### Linking research perspectives to action: practical relevance

The collaborative research effort we initiated and structured will likely contribute to more impact and higher quality of research, supported by theory, through the structural exchange of expertise and opportunities for focus when applying for external funding. The research agenda has been communicated and will be utilized in a policy process to increase the likelihood of this outcome. Our study, particularly the participatory approach, can serve as a model for other HPE researchers to establish shared research objectives; it initiates successful collaborations, enhancing accountability and transparency towards stakeholders. Professionals involved in policy implementation can identify necessary conditions for successfully implementing research collaboration initiatives between researchers in different academic organizations.

### Strengths and limitations

We have developed a research agenda through a thorough methodology and an intensive process of support and collaboration. Nevertheless, in presenting this research agenda and our considerations regarding collaboration, we only cover the first step of research collaboration between researchers in different organizations. Because we take the stance in our work of a living research agenda—not a finished blueprint but a tool that could support the development in working together—we expect the next steps to follow (Kezar, 2005). Further collaboration on a positive footing is also to be expected because this research collaborative network evolves in the broader context where funding agencies, who subscribed to our point of departure of increasing quality and impact through collaboration, opened a call for funding which would be only granted if we arranged and build upon further collaboration.

A disadvantage of the Q-methodology is that it is impossible to determine whether the concourse is complete. The analyses occur based on the ordering of the 64 statements that were provided to the respondents. In theory, it is always possible that critical perspectives are missed because certain items were not presented in the concourse (Leidig et al., 2022). In our case, this is possible but not likely. We compiled the set of items with a group of GP-HPE researchers with a wealth of experience in this field as researchers, educators, GPs, and education developers. Participants’ statements during the interviews would not have changed the concourse significantly.

Finally, the interviews after the sorting were brief because of time limitations during individual sorting in a group meeting. As a result, not all questions in the format for the interviews were answered during the interviews. This data was not analyzed separately but proved valuable as we used them during the qualitative analysis of the holistic ordering of the statements.

### Future research

Our study provides perspectives on GP-HPE research. When, in the future, learning and training within the General Practice domain will always occur interprofessionally, the P-set of a Q-sorting method should include the patient voice as well as those of nursing and other allied health professionals in setting the research agenda. In addition, there is still a need for more insight into which studies in this domain are missing, which research findings are of insufficient quality, and which aspects may need further attention. This is, as we argued in the introduction, a time-consuming undertaking in our broad interdisciplinary field. Further literature review on each of the perspectives will help to tackle this task incrementally; it will support the definition of evidence gaps within each perspective.

## Conclusion

This paper reports an empirical study creating a research agenda that provides a national collaborative focus on GP-HPE research in the Netherlands. We found five different stakeholder perspectives on topics that need further scientific research. Additionally, we took a reflexive stand during the process and, as a result, brought conditions to the surface for successful national collaboration. One of these conditions was using the Q-methodology in each institute which built trust among researchers of separate institutes. Our approach to setting a research agenda connects two ideas that may usually be considered separate. First, in the way it aligns academic and non-academic perspectives; second in how the process helped to develop directions for reaching collaborative advantage through inter-institutional competition. This research project might set an example for HPE researchers worldwide aiming for collaboration to improve the evidence base in their field.

### Supplementary Information

Below is the link to the electronic supplementary material.

Supplementary file1 (DOCX 74 kb)
