# Supplementary material for: Advancing collaboration in health professions education in the general practice domain, developing a national research agenda
Source: Adv Health Sci Educ Theory Pract. 2024 May 27;29(4):1417–34. doi: 10.1007/s10459-024-10340-4 (PMC11369045; doi:10.1007/s10459-024-10340-4)
Supplement: Supplementary file 1 — Supplementary file1 (DOCX 74 kb) [file 10459_2024_10340_MOESM1_ESM.docx]

Advancing collaboration in Health Professions Education
in the General Practice domain:
developing a national research agenda

Appendix

**Information participants received at the start of the session**

At the start of each Q-sorting exercise, the facilitator of that session gave a brief presentation. The presentation gave some background on the rationale for the agenda: A broad multidisciplinary field, the wish to cooperate more, and the idea that a shared agenda could support that process. In addition, participants were told that their data would be anonymized and that the coding (who is behind each code) would be stored separately from the data. In order to know more about in what role they took part, one question was asked on their background.

Participants were informed that the statements did not reflect research questions but general topics for research, focusing on content, learning approach, or context).

Finally, participants were recommended (based on our experiences during the pilot session) to start reading the explanations in different order to prevent that in each group statement 1 was understood very well, but statement 64 less so. Participants were not required to read all explanations before starting to sort, as some statements may be self-evident for them while others are perhaps less so. Participants were invited to write everything that stood out for them on their documents. In practice, this was done by just a few participants; therefore, this was not considered a data source.

Participants were given the following information at the start of the Q-sorting session:

- An A4 document with the central question they had to keep in mind during the session: “Which topics should be included in the research agenda for the continuum of learning and supervision of General Practitioners in the Netherlands, because you think that research is necessary on those topics in the next six years?”
- An overview of each of the 64 statements with an explanation of what was meant with each statement
- An A4 document with an empty Q sort grid on which their code was written
- A stack of 64 laminated cards with a statement on it
- An A3 size Q sort grid on which they could order their statements
- Documents for ethical consent as mentioned above.

**Manner of involvement**

Each participant was asked to answer the following question:

In reporting the results it is important to make it clear in what manner you have been involved in the topic of this Q sort session. Which of the different manners of involvement are applicable to you? More than one may apply.

- Participated in educational research
- Performed educational research
- Applied results of educational research in the design of my teaching activities
- Applied results of educational research in the way I teach

**Interview questions**

1. What reasons did you have for putting this statement (pointed at during the interview) at the ultimate right side of the grid (“include certainly +6)
2. What reasons did you have for putting this statement (pointed at during the interview) at the ultimate left side of the grid (“include certainly not +6)
3. Can you explain why you put this statement (number of the statement) here? (if applicable, when a statement is standing out because of the position of other statements)
4. I saw that you were in doubt about the position of this statement (if applicable), can you tell more about this?
5. I saw …{something special, for example laughing}…when reading this statement (if applicable), can you tell more about this?
6. Are there other things that you want to share about this Q-sorting session? In general or related to the statements?
7. Did you miss certain topics in the list of 64 statements?

**Translated statements used in the Q-sorting**

| 1 | Clinical reasoning | Learning to clinical reasoning in different contexts |
| --- | --- | --- |
| 2 | Professionals in healthcare as researcher | Learning to become, be, and remain a researcher. Contribution of these researchers to the knowledge of the profession |
| 3 | Ethical reasoning | Learning to reason ethically |
| 4 | Cultural competence in organization | Learning to work in a safe, effective and respectful way in organizations within a context of cultural diversity with colleagues of different generations (not: The patient in the consulting room) |
| 5 | Continuity of care | Learning how, as a GP, you can take and feel personal responsibility for your patients and assist them for a longer period of time. In this, the balance between personal and team continuity is important as well as continuity of information |
| 6 | Patient-centered care | The process and outcome of (different) ways of learning to deliver patient-centered care |
| 7 | Dignity and autonomy for patients | Learning to ensure dignity and autonomy for patients, especially when patients are from other cultures |
| 8 | Uncertainty | Learning to deal with clinical uncertainty |
| 9 | Width of the profession | Learning to deal with the tension between a broader definition of health and learning the (biomedical) basics |
| 10 | Evidence-based care | Learning to deliver evidence-based care |
| 11 | Professionality of the GP | The impact of education on how the AIOS develop as a professional within healthcare dynamics |
| 12 | Management | Learning to function within a (GP organization) at management level, possibly making them more willing and able to become a practice owner, for example |
| 13 | Triage | Learning to ensure safe and efficient triage in the General Practice and fulfil the role as a steering physician |
| 14 | OneHealth | Learning about issues dealing with the relationship between human and animal pathologies, whether or not in collaboration with professionals (including veterinarians) dealing with zoonosis |
| 15 | Professional Identity | How healthcare professionals develop a professional identity and how this development process can be/is influenced by activities at the level of clinical practice and education |
| 16 | Clinical skills | Different ways of learning diverse clinical skills |
| 17 | Communication skills | Learning to communicate with patients in different cultural contexts |
| 18 | (Regulate) Emotions | Learning how to regulate your own emotions and acquire emotional intelligence for different situations in education and at work |
| 19 | Patient safety | Learning to perceive aspects of patient safety and deal with them in one's own (clinical) organization, including factors that inhibit or stimulate it. |
| 20 | Decision making | Learning to make decisions, both individually, together with the patient, and in groups of professionals |
| 21 | Diversity in patient care | Learning to provide care to a diverse patient population and to deal with various health issues (e.g. transsexuality, mental problems, addictions). |
| 22 | Transitions | Learning to deal with change in a broad sense. E.g. from basic to advanced training. |
| 23 | Interprofessional teamwork | Learning to cooperate interprofessionally and multidisciplinary in healthcare. This involves working with people who all have a (para)medical background. |
| 24 | Community oriented care | Learning to work together interprofessionally and multidisciplinary in the community. This involves cooperation with people who do not all have a (para)medical background (e.g. police) |
| 25 | Agency | Learning to shape the profession of the future, locally, nationally and internationally. Understanding different modes of leadership and understanding the relationships between different parts of the healthcare |
| 26 | Planetary Health | Learning to practice the profession for the broader context of health e.g. in the impacts of climate, safety, pollution |
| 27 | Innovation of healthcare | Learning to contribute to healthcare innovation |
| 28 | E-health and other digital innovations | Learning to understand and use innovations |
| 29 | Multimedia communication | Learning to communicate through phone, video link etc. |
| 30 | Life-long learning | Learning that takes place after completion of undergraduate and post-graduate education which makes knowledge and skills of themselves or a group up-to-date |
| 31 | Learning environment | Promote a positive learning culture within undergraduate and postgraduate education, where, for example, learning from mistakes and mentoring and supervision take place. Plus what effect this has on shared values, knowledge and practices of learners. |
| 32 | Unsafety learning culture | Prevent an unsafe organizational culture within undergraduate and postgraduate education (including places for clerkships). Influencing factors of psychological and physical well-being and how to deal with negative behavior are also included. |
| 33 | Entrepreneurship | Learning to deal entrepreneurially and contribute to changing practice management in General Practice interpersonal (couples, health centers, team of General Practice, multiple trainees in a practice) |
| 34 | Combination of roles in General Practice | Learning how to deal with and contribute to (new) combinations of different roles by GPs (clinician-teacher e.g.) within undergraduate and postgraduate education (including training places) |
| 35 | Regionalization | Regionalization of undergraduate and postgraduate education (including training places) by stimulating cooperation between GP training institutes and what is needed in a specific region (macro) |
| 36 | Networked healthcare | Learning to deal with and contribute to network medicine (way of working in clinical practice, the network is always multidisciplinary and/or interprofessional) during undergraduate and postgraduate education (including training places) |
| 37 | International education | Compare similarities and differences between courses in different countries, with the aim of making courses more compatible |
| 38 | Patient involvement | Forms of teaching and learning processes when real (not simulated) patients are involved in education |
| 39 | Technology enhanced learning | Forms of teaching and learning processes when technology is used. Also: When physical, when digital |
| 40 | Portfolio learning | Forms of teaching and learning that take place in situations where portfolios are used |
| 41 | Observational learning | Forms of teaching and learning processes when AIOS/supervisors and/or by AIOS/supervisors observe each other |
| 42 | Networked learning | Forms of teaching and learning that take place in networks, often online |
| 43 | Workplace learning | Learning on workplace with the aim to learn to function in the workplace during that teaching, but may also have other aims (more substantive). |
| 44 | Learning in inter professional setting | Learning in a setting with multiple, different disciplines or professionals. Goal is to learn interprofessional cooperation during that education |
| 45 | Self-regulated learning | Learning through (new) forms of SRL (self-regulated learning). |
| 46 | Learning from Experience | Learning from authentic experiences. Purpose of that learning can be different, e.g. empathy or negotiation. |
| 47 | Multi-level learning | Learning in a setting with different levels of learners (student, fellowship, AIOs, HA, trainer) |
| 48 | Peer-assisted learning | Learning in a setting with similar levels |
| 49 | Learning by socialisation | Learning through the implicit influence that exists in a particular organization or culture (unwritten rules, rituals, or patterns of desired behavior) that learners adopt. |
| 50 | Interdisciplinary education | Involving professionals and content from other disciplines, e.g. medical humanities or linguists, in education |
| 51 | Workplace assessment | Tools to assess learners in the workplace and about the environment in which these assessment tools are used |
| 52 | Programmatic assessment | Programmatic assessment to look at the whole development of the student. The aim is both to optimize the reliability of decisions and to stimulate the student's learning process |
| 53 | Relation between assessment and motivation | Assessment in relation to motivation during different forms of learning and how it balances social accountability, summative decision-making and motivation |
| 54 | Cost-effectiveness of education | Cost-effectiveness of educational programmes and supporting resources (such as e-learning) |
| 55 | Differences in GP education | Differences and similarities in GP education by comparing the Netherlands with other countries for the benefit of international cooperation |
| 56 | Faculty development | Continuing medical education (CME) and continuous professional development (CPD) for teachers and trainers |
| 57 | Curriculum evaluation | Evaluation of curricula to ensure to deliver graduates who can work well in different local contexts (country, region). Including, in the Netherlands, alignment with Gear and Nivel evaluation. (Macro) |
| 58 | Remediation | Policy(s interventions) to help students who have problems completing the programme to make responsible choices. Also around professionals who do not perform well and where education can provide a possible solution. |
| 59 | Relation between workplace learning and institute learning | Policy and interventions to get the relationship between workplace learning and institute learning right. Organize relationships in the student/AIOS-teacher-trainer triangle optimally |
| 60 | Relation between undergraduate education and postgraduate education | Policy and interventions to ensure a good fit between bachelor-master (undergraduate) education and GP postgraduate programs (macro) |
| 61 | Selection of learners | Policies and interventions around selection and entry processes in GP training (macro) |
| 62 | Carriere choice | Policy and interventions around students' choices for (continuing) education, including the choice of undergraduate students for continuing education (macro) |
| 63 | Flexibility | Policies and interventions around the flexibilization of training, for example when students or AIOS already have certain baggage that allows them to get exemptions |
| 64 | Subjectification | Policies and interventions to contribute in GP training to subjectification as part of professional development (in addition to and with qualification and socialization). With subjectification, the AIOS himself takes responsibility within the profession |
|  |  |  |

**Grid**

Code respondent: ……

| -6  Include certainly not | -5 | -4 | -3 | -2 | -1 | 0  Neutral | +1 | +2 | +3 | +4 | +5 | +6  Include certainly |
| --- | --- | --- | --- | --- | --- | --- | --- | --- | --- | --- | --- | --- |
|  |  |  |  |  |  |  |  |  |  |  |  |  |
|  |  |  |  |  |  |  |  |  |  |  |  |  |
|  |  |  |  |  |  |  |  |  |  |  |  |  |
|  | |  |  |  |  |  |  |  |  |  |  | |
|  | | |  |  |  |  |  |  |  |  | | |
|  | | | |  |  |  |  |  |  | | | |
|  | | | | |  |  |  |  | | | | |
|  |  |  |  |  |  |  |  |  |  |  |  |  |
|  | | | | | |  |  | | | | | |
|  |  |  |  |  |  |  |  |  |  |  |  |  |
